# Locoregional Management of Breast Cancer: A Chronological Review

**DOI:** 10.3390/curroncol29070369

**Published:** 2022-07-01

**Authors:** Abdulla Al-Rashdan, Melina Deban, May Lynn Quan, Jeffrey Q. Cao

**Affiliations:** 1 Dalhousie University School of Medicine, Dalhousie University, Halifax, NS B3H 1V7, Canada; ab613894@dal.ca; 2 Cumming School of Medicine, University of Calgary, Calgary, AB T2N 1N4, Canada; melina.deban@albertahealthservices.ca (M.D.); maylynn.quan@albertahealthservices.ca (M.L.Q.); 3 Division of Radiation Oncology, Tom Baker Cancer Centre, 1331 29 St. NW, Calgary, AB T2N 4N2, Canada

**Keywords:** breast cancer, locoregional, management, axilla, surgery, radiotherapy

## Abstract

Locoregional management of breast cancer is founded on evidence generated over a vast time period, much longer than the career span of many practicing physicians. Oncologists rely on specific patient and tumour characteristics to recommend modern-day treatments. However, some of this information may not have been available during prior periods in which the evidence was generated. For example, the comprehensive Early Breast Cancer Trialists’ Collaborative Group (EBCTCG) meta-analyses published in the 2000s typically included older trials accruing patients between the 1960s and 1980s. This raises some uncertainty about whether conclusions from studies conducted in prior eras are as relevant or applicable to modern-day patients and treatments. Reviewing the chronological order and details of the evidence can be beneficial to understanding these nuances. This review discusses the evolution of locoregional management through some key clinical trials. We aim to highlight the time period in which the evidence was generated and emphasize the 10-year outcomes for the comparability of results. Evidence supporting surgical management of the breast and axilla, as well as details of radiotherapy are discussed briefly for all stages of breast cancer.

## 1. Introduction

**Figure 1:**
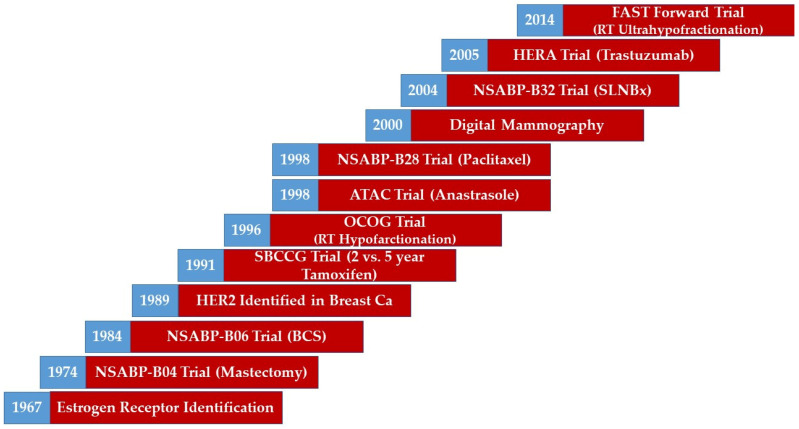
A timeline illustrating the evolution of breast management. Abbreviations: BCS, Breast Conserving Surgery; CA, Cancer; HER2, Human Epidermal Growth Factor 2; RT, Radiotherapy. Note: This figure represents a broad overview of the evolution of breast management. However, it does not represent the exact time of the adoption of the measure in the trial. Trials are reported by end accrual dates.

Breast cancer management has evolved over time with notable advances and milestones [1,2,3,4,5,6,7,8,9,10,11,12,13,14,15]. Advances in radiological imaging have refined cancer detection and localization [1]. Enhanced pathological processing and tumour profiling have yielded a more detailed characterization of disease [2,3]. Advances in surgical techniques and approaches have allowed for breast conversation and minimization of morbidity [4,5,6,7,8]. More effective systemic treatment options have resulted in improved survival outcomes [9,10,11,12,13]. Modern radiotherapy techniques have enabled precise dose delivery while minimizing dose to organs at risk [14,15]. Examples of the advances and milestones of breast cancer management over time can be seen in Figure 1.

Breast cancer trials that define modern-era treatment approaches have varied based on patient (e.g., age), tumour (e.g., receptor status), and treatment (e.g., type or sequence) characteristics. Locoregional management decisions are based on evidence at various time periods with improved understanding of patient and tumour characteristics and systemic treatments evolving over time. In this paper, we review the key randomized controlled trials (RCT) over time (based on accrual dates) contributing to the locoregional management of generally defined risk groups of patients with breast cancer. We emphasize the 10-year outcomes, whenever reported, to facilitate comparison between various trials.

## 2. Early Stage, Low Risk (T1-2N0-N1mic)

### 2.1. Mastectomy

Total mastectomy (TM) is usually curative for early stage breast cancer patients with outcomes similar to radical mastectomy (RM). The National Surgical Adjuvant Breast and Bowel Project (NSABP) B-04 clinical trial (1971–1974) randomized patients to RM, TM without axillary clearance but with locoregional radiotherapy (TMR), and TM alone. The 10-year local recurrence (LR) rates for those with negative axilla were 4.4% vs. 1.1% vs. 7.7%, respectively. There was no difference in disease-free survival (DFS) or overall survival (OS). Overall survival was 25%, 19%, and 26%, respectively (*p* = 0.68) [6]. The Early Breast Cancer Trialists’ Collaborative Group (EBCTCG) meta-analyses (2005), which included trials with reported results between 1985 and 2000, showed that the 10-year isolated LR after TM with axillary clearance and without radiotherapy (RT) was 8.0% and the breast-cancer-specific mortality (BCSM) was 20.8%. The effect of RT on LR and BCSM was modest for node-negative disease at 3.1% and 22.3%, respectively [16].

### 2.2. Breast-Conserving Surgery

Breast-conserving surgery (BCS) was compared with TM in trials that accrued patients between 1976 and 1991. The NSABP B-06 trial (1976–1984) compared patients with tumours ≤ 4 cm into one of three arms: TM alone, BCS + whole breast radiotherapy (WBRT), and BCS alone. The DFS of the three arms was 51 vs. 50% vs. 48%, *p* = 0.15, respectively, and the overall OS was 62% vs. 62% vs. 60%, *p* = 0.77, respectively [7]. The 12-year LR rates reported for patients with node-negative disease in the BCS + RT vs. BCS alone arms were 12% vs. 32%, *p* ≤ 0001, respectively. At 20 years of follow-up [17], ipsilateral breast tumour recurrence (IBTR) was 14.3% for women who had undergone BCS + WBRT vs. 39.2% for BCS alone (*p* < 0.001). The benefit associated with radiation was independent of nodal status. No difference was noted in disease-free survival (36% vs. 35% vs. 35%, respectively, *p* = 0.26) and overall survival (47% vs. 46% vs. 46%, respectively, *p* = 0.57). Criticisms of the study include the description of recurrences in the BCS group as “non-events”. Patients randomized to BCS received intraoperative frozen section margin assessment; if the margins were positive, they were converted to a total mastectomy. In the intention-to-treat analysis, they were included in the BCS group. Lastly, only node-positive women received adjuvant systemic therapy, differing from modern-day regimens.

The Milan Cancer Institute trial (1973–1980) randomized 701 women with tumours of 2 cm or less to RM or quadrantectomy (BCS) with WBRT. The 20-year update showed LR of 8.8% for women who underwent BCS with WBRT and 2.3% for the RM group (*p* < 0.001). There was no statistically significant difference in terms of contralateral breast cancer, distant metastases, or other primary cancers. Overall survival was 41.7% vs. 41.2%, respectively (*p* = 1.0) and breast-cancer specific survival was similar (*p* = 0.8). Despite increased LR rates in the context of dated radiotherapy techniques, this trial was able to demonstrate that BCS did not reduce survival [18].

Nearly a decade later, the National Cancer Institute (NCI) led a trial (1979–1987) with 237 women with stage I and II breast cancer. They were randomized to modified radical mastectomy (MRM) or BCS with ALND and WBRT. The proportion of patients with tumours ranging between 4.1 and 5 cm were 9% and 7%, respectively [19]. Comparatively, in the Milan trial, close to 50% of the tumours measured less than 1 cm in size. NCI allowed macroscopic tumour excision and a second surgery to achieve adequate margins. After 18 years of follow-up, LR occurred in no patients with MRM and 27 patients with BCS. A total of 16 of these patients (59%) was salvaged successfully with mastectomy, while the remaining failed regionally and distantly. There was no significant difference in disease-free survival (67% vs. 63%, *p* = 0.64) and overall survival (58% vs. 54%, *p* = 0.67). This trial has been criticized for its definition of adequate margins, which may have contributed to the observed LR rate.

The EBCTCG meta-analysis (2005) highlighted that BCS alone is associated with a high 10-year isolated LR (29.2%) in patients with node-negative disease [16]. However, in more recent trials [20] with follow-up after 2006, the LR rates are much lower compared to the earlier studies included in the EBCTCG meta-analysis (2005). Reasons for this reduction include the evolution of systemic treatment options that reduced recurrences, improvement in diagnostic techniques for better tumour characterization, and improvement in pathological assessment techniques [21].

The question whether BCS alone is adequate treatment in patients with a very low risk of recurrence has been raised in a couple of well-known RCTs and today remains an interesting study question with advances in genomic expression profiling. The Cancer and Leukaemia Group (CALGB) 9343 trial (1994–1999) enrolled low-risk patients defined as those 70 years of age or older with small tumours (primarily ≤ 2 cm); Estrogen Receptor (ER)-positive were planned to receive 5 years of Tamoxifen [22]. Patients were randomized to adjuvant whole breast RT vs. BCS alone. Interestingly, 53% of the population survived at least 10 years. The 10-year LR rate after BCS alone with tamoxifen was 10% compared to 2% for BCS + WBRT. There was no difference in breast-cancer-specific survival (BCSS) or OS. The PRIME II trial (2003–2009) was a similar study, but included a slightly younger population (age > 65 years) and patients had slightly larger tumours (≤3 cm). The trial allowed either grade III or lymphovascular invasion, but not both. Patients would also receive 5 years of endocrine treatment. The 10-year LR was 9.8% in BCS alone compared to 0.9% in BCS with WBRT (*p* < 0.0001). OS was 80.4% vs. 81%, *p* = 0.68, respectively [23]. Unfortunately, neither of these trials included a radiation therapy alone arm given the marginal absolute benefit of endocrine therapy in this older patient population. However, Ward et al. [24] constructed a Markov model to estimate the 10-year risk of IBTR with RT alone and reported to be 4.24%. There are numerous active low-risk biomarker-based studies exploring the role of BCS and ET alone: LUMINA (ClinicalTrials.gov identifier: NCT01791829, accessed on 15 April 2022), The IDEA Study (NCT02400190), The PRECISION Trial (NCT02653755), PRIMETIME [25], and EXPERT (BIG 16-02/ANZ 1601) [26], which have since completed accrual with imminent results that will help to guide patient selection and clinical treatment decision making.

### 2.3. Axillary Surgery

#### 2.3.1. Clinically Node-Negative

Axillary lymph node dissection (ALND) provided both diagnostic and therapeutic approaches in older trials. However, ALND is associated with significant morbidity, such as lymphedema, arm paraesthesia, and lower quality of life [27]. Further, only about a third of patients with clinically negative axilla will have pathological nodal involvement. Thus, ALND is overtreatment for many women with clinically negative axilla [8], which has resulted in the current-day standard of care sentinel lymph node biopsy (SLNB).

The feasibility of SLNB was described by Krag et al. [28]. In their multicenter validation study, 443 patients with breast cancer underwent SLNB with technetium-99 m sulphur colloid followed by a completion axillary dissection. The successful identification of the sentinel lymph node (SLN) was achieved in 93% and the accuracy was 97%. Sensitivity and specificity were reported at 89% and 100%, respectively. The false-negative rate (FNR) was 11%, mostly in the lateral half of the breast on univariate analysis (*p* = 0.004).

The landmark ALMANAC trial (1999–2003) accrued 1031 patients to be randomized to the following arms: SLNB or standard axillary surgery. Patients with positive SLNs were then treated with axillary surgery or radiotherapy, depending on their local protocol. SLN localization was successful in 98% of patients. At 12 months, SLNB reduced by 63% the risk of lymphedema (95% CI 0.23–0.60) and sensory loss (95% CI 0.27–0.50). The length of hospital stay, drain usage, return to normal activities, arm function, and quality of life were all superior in the SLNB arm [29].

NSABP B-32 trial (1999–2004) compared patients with clinically negative axilla who had SLNB with immediate ALND to those with SLNB alone [8,30]. Patients were treated with BCS and mainly had tumours ≤ 2 cm. SLNB identification rate was 97% and the false-negative rate was 10%. Approximately 98% of SLNB were in axillary levels I–II. The 8-year regional recurrence (RR) rate was 0.4% vs. 0.7%, *p* = 0.22, respectively; DFS was 82.4% vs. 81.5, *p* = 0.54, respectively; and OS was 91.8% vs. 90.3%, *p* = 0.12, respectively.

Further evidence supporting the omission of axillary surgery in patients with very low-risk disease and clinically negative axilla comes from the CALGB 9343 trial [22], where 63% of the population did not have axillary surgery. Axillary failure rates were 3% in BCS alone vs. 0% in BCS + RT group. This was comparable to patients in PRIME II trial [23], where all patients underwent either SLNB or ALND; the RR rates were 2.3% in BCS alone vs. 0.5% in BCS + RT group (*p* = 0.014).

#### 2.3.2. Clinically Node-Negative Prior to Neoadjuvant Therapy

SLNB can be an appropriate diagnostic approach for patients with clinically negative axilla who receive neoadjuvant chemotherapy. In NSABP B-27 trial (1995–2000), several surgeons performed a SLNB prior to the required ALND after neoadjuvant therapy [31]. Their results were published in 2005 by Mamounas et al. [32]. Out of the 2411 patients randomized for B-27, 428 had lymphatic mapping with available operative and pathology reports. Compared with patients who did not undergo SLNB attempt, these patients had smaller tumours (*p* = 0.002) and 76% had no involvement of axillary lymph nodes (*p* = 0.002). A radioactive colloid was used in 15%, blue dye in 30%, and both in 55% (remaining with unclear method). In the overall cohort, the rate of success in identifying an SLN was 84.8% and 84.4% in patients who were clinically node-negative. The rate of identification was similar for radiocolloid (88.9%) and the combination (87.6%), but lower for blue dye alone (78.1%) (*p* = 0.03). Overall accuracy was reported at 95.6% and FNR was 10.7%. A recent meta-analysis [33] showed that the SNLB identification rate was 96% (95% confidence interval (CI): 95–97%) with false-negative rate of 6% (95% CI: 3–8%).

#### 2.3.3. Micrometastatic Disease

While node-negative patients do not require axillary dissection if SLNB is negative, those with micrometastatic disease seem to have comparable outcomes when undergoing observation as opposed to ALND [34,35]. The International Breast Cancer Study Group (IBCSG) 23-01 trial (2001–2010) [34] enrolled patients with tumours ≤ 5 cm and with lymph node micrometastasis or isolated tumour cells on SLNB, and were randomized to ALND vs. no further axillary surgery. The recent update showed 10-year RR for ALND vs. observation were 1% vs. 2%, respectively; DFS was 74.9% vs. 76.8%, *p* = 0.24, respectively; and OS was 88.2% vs. 90.8%, *p* = 0.2, respectively.

### 2.4. Adjuvant Radiotherapy

#### 2.4.1. Conventional Fractionation WBRT

Radiotherapy to the whole breast has provided a pivotal role in reducing local recurrence and improving survival compared to BCS alone. Earlier studies that established the benefit of RT used conventional RT doses of 45–50 Gy delivered in 25 fractions with some allowing a boost to the tumour bed. The EBCTCG meta-analysis (2005) showed that RT reduced 10-year LR by 19%, which translates into a 2.9% reduction in 10-year BCSM (*p* = 0.006) [16]. Of note, many studies included in the meta-analysis did not allow tamoxifen in the BCS alone arm and chemotherapy treatment options were older, non-anthracycline-containing regimens.

#### 2.4.2. Hypofractionation WBRT

Hypofractionation presented an opportunity to deliver treatment in a shorter time interval. Dose fractionation in these whole breast trials were 2.25–3.53 Gy/fraction delivered in 13–20 fractions. The most common hypofractionation regimens were those with cumulative doses of 40–42.5 Gy delivered in 15–16 fractions. The Ontario Cooperative Oncology Group (OCOG) trial (1993–1996) included patients with tumours ≤ 5 cm. The 10-year LR was 6.7% vs. 6.2%, respectively. DFS and OS were similar in both groups (87%, and 84%, respectively) [36]. The START B trial (1999–2001) accrued women with tumours ≤ 3 cm, and most received adjuvant systemic treatment. The 10-year LR was (3.8% vs. 5.2%; *p* = 0.1), while the estimated all-cause mortality was lower by 3.3%, *p* = 0.042; both were in favour of the hypofractionated arm [37].

Toxicity was similar in the OCOG study arms. The START B trial [37] showed that moderate or marked breast shrinkage (26.2% vs. 31.2%; *p* = 0.015), telangiectasia (4.2% vs. 5.8%; *p* = 0.03), and breast oedema (5.1% vs. 9%; *p* = 0.001) were lower in the hypofractionated arm compared to the conventional RT arm.

#### 2.4.3. Accelerated Partial Breast Irradiation

The observation that a significant proportion of LR after BCS were in proximity to the tumour bed [7] led to the thought of radiotherapy de-escalation in the form of accelerated partial breast irradiation (APBI), instead of whole breast irradiation (WBI), might result in a comparable tumour control with a potentially a better toxicity profile. Further, it represented a potential solution for delivering treatment in even shorter intervals than hypofractionation.

Trials examining APBI accrued patients between 1998 and 2013 evaluating multiple techniques. In this review, we only focus on key trials using external beam radiotherapy solely with partial breast irradiation. The NSABP B39-RTOG 0413 trial (2005–2013) [38] and Canadian RAPID trial (2006–2011) [39] compared APBI delivered in an accelerated fashion through three-dimensional conformal radiotherapy (3DCRT), 3.85 Gy/fraction, twice daily for 5 days to WBI, while the University of Florence trial (2005–2013) compared intensity-modulated radiotherapy (IMRT) delivered in 6 Gy/fraction, every other day over 5 fractions to WBI [40].

The 10-year LR for NSABP B39-RTOG 0413 was 3.9% in WBI compared to 4.6% in the APBI arm. The trial primary endpoint (equivalence) was not met statistically, but considered equivalent clinically. The 10-year recurrence-free interval favoured WBI (93.4%) vs. APBI (91.8%), *p* = 0.02, but with no difference in OS (91.3% vs. 90.6%; *p* = 0.35). The 8-year LR for RAPID was 2.8% for WBI vs. 3% for APBI, which met statistical significance for non-inferiority. The 10-year LR in the University of Florence was 2.5% in WBI vs. 3.7% in APBI. Reasons for low recurrence rates include conservative eligibility criteria to include low-risk patients; however, there were some node-positive patients enrolled. Further, the trials included a subset of patients with ductal carcinoma in situ and enrolled patients in an era of modern systemic treatment. It is noteworthy that the NSABP B39-RTOG 0413 included patients younger than 40 years, GIII disease, and more patients with tumours > 2 cm in size.

Toxicity reporting varied among the three trials. The NSABP B39-RTOG 0413 reported lower GII or higher toxicity in the APBI arm. The RAPID trial reported better GII or higher acute toxicity at 3 months 45% vs. 28%, *p* < 0.0001. However, late toxicity was worse for APBI with the 5-year cumulative incidence was 13% vs. 32% (*p* < 0.0001). Toxicity was mainly breast induration (4.6% vs. 22.9%) and telangiectasia (3.7% vs. 9.3%). Conversely, the University of Florence trial showed better cosmesis of APBI. The late GII toxicity or higher was 2.7% vs. 0%; *p* = 0.015.

When reviewing cosmesis outcomes between arms and trials, it is worthwhile and important to note the control arm use of conventional versus hypofractionation. Future studies should be focused on APBI in five daily fractions compared to ultrahypofractionation, which will be elaborated upon in the next section.

#### 2.4.4. Ultrahypofractionation

Ultrahypofractionated WBRT, defined as regimens with ≥5 Gy per fraction, was tested in the FAST-Forward trial (2011–2014), which compared 40 Gy delivered in 15 fractions to 27 Gy and 26 Gy delivered in 5 daily fractions. The LR at 5 years was 2.1% vs. 1.7% vs. 1.4%, respectively, with no difference in survival. The clinician-assessed, moderate-marked adverse effects at 5 years were 9.9% vs. 15.4% vs. 11.9%, respectively [41]. Due to the comparable results at 5 years and in light of the COVID-19 pandemic, several programs adapted the FAST-Forward regimen of 26 Gy to minimize the possibility of exposure to the virus and minimize the possibility of treatment interruption [42]. The key trials in low-risk breast cancer can be seen in Table 1.

## 3. Intermediate Risk (T2N1, T3N0)

### 3.1. Mastectomy

Mastectomy represents a treatment option for patients with intermediate-risk breast cancer. Recurrence and survival have improved over the years. The EBCTCG meta-analysis (2014) showed that the 10-year locoregional recurrence (LRR) and BCSM in patients with N1 disease who were treated with mastectomy and axillary surgery and without RT were 20.3% and 36.8%, respectively [43]. However, this meta-analysis included older trials accruing patients between 1964 and 1986. Additionally, 86% of patients were in trials where the systemic treatment was either Tamoxifen or cyclophosphamide, methotrexate, and fluorouracil (CMF). In contemporary series where a modern-day systemic therapy is used, the LRR is lower in the range of 3–9% [44,45].

### 3.2. Breast-Conserving Surgery

Upfront BCS is a reasonable option for patients with T2N1 disease, despite being at a higher risk of LRR compared to those with node-negative disease. Almost one-third of the population of the NSABP-B6 trial [7] with N1 disease had higher LR compared to those with node-negative disease. This is confirmed in the EBCTCG meta-analysis (2005) as those with BCS alone had 10-year LR in node-negative (29%) vs. node-positive disease (46.5%) [16].

In the Danish randomized DBCG-82TM trial (1983–1989), 731 women less than 70 years were correctly randomized to BCS and ALND or modified radical mastectomy (MRM) and evaluable for recurrence and survival outcomes [46]. Remaining patients either declined randomization or were subject to randomization error. In contrast with the studies mentioned earlier, tumours of any size were allowed and the mean tumour diameter was 18 mm in both groups. Radiation was administered to all BCS patients (with a boost of 10–25 Gy) and to women with MRM and positive nodes. At a median of 20 years of follow-up, local recurrence was observed in 13% of patients with BCS vs. 21% of patients with MRM. The 10-year recurrence-free survival (60% vs. 61%, *p* = 0.57) and 20-year overall survival (58% vs. 50%, *p* = 0.20) were not significantly different between both groups.

### 3.3. Axillary Surgery

Several studies have generated a substantial debate on whether ALND is needed for clinically node-negative patients who have minimal axillary involvement after SLNB. Advancement in pathological specimen evaluation by immunohistochemistry (IHC) has led to the identification of positive nodes that would have been considered negative in patients treated in the 1980s. This leads to the speculation that some of those labelled with N1 in the contemporary series might have a more favourable prognosis than the historical N1 disease [47]. Advances in sonographic imaging nowadays take into consideration changes in cortical thickening, shape, or vascular pattern as well as hilum decrease or absence to trigger biopsies, and potential upstaging in patients would have been considered clinically node-negative on routine physical examination.

The American College of Surgeons Oncology Group (ACOSOG)-Z0011 trial (1999–2004) compared ALND to no axillary intervention in patients with clinically negative axilla and found to have 1–2 positive lymph nodes on SLNB. The trial mandated that lymph node identification was based on haematoxylin and eosin staining and excluded those identified with IHC [48]. Radiotherapy was intended to the breast only, although a review of the detailed radiotherapy plans of a subset of patients showed that 51% were treated with high-tangents and that 15% received a supraclavicular field [49]. There was no difference in the 10-year cumulative nodal recurrence (0.5% vs.% 1.5%; *p* = 0.28).

The SINODAR One study was a prospective, noninferiority, multicentre Italian study that was started in April 2015 to further clarify the role of axillary lymph node dissection in patients with T1 or T2 breast cancer with 1 or 2 macrometastatic lymph nodes undergoing either breast conserving surgery or mastectomy [50] to confirm the results of the Z0011. The early results were shared at SABCS 2021 with median follow-up at 34 months with one axillary recurrence in each arm (*p* = 0.489) and no significant differences with respect to OS or recurrence-free survival.

The European Organisation for Research and Treatment of Cancer (EORTC) 10,981–22,023 AMAROS (After Mapping of the Axilla, Radiotherapy or Surgery) trial (2001–2010) compared ALND to axillary RT alone. A total of 95% of the accrued patients had metastasis limited to 1–2 positive lymph nodes. The 5-year axillary recurrence was 0.4% vs.1.19%, respectively; DFS was 86.9% vs. 82.7%, *p* = 0.18, respectively; and OS was 93.3 vs. 92.5, *p* = 0.34, respectively [51]. The long-term update was provided at the San Antonio Breast Cancer Symposium (SABCS) in 2018, showing that, after 10 years, 1.82% (11/681) assigned to axillary RT had axillary recurrence, compared with 0.93% (7/744) with ALND. It should be noted that AMAROS allowed for mastectomy, but only accounted for 17% of all patients. Further, approximately 40% of all patients had only isolated tumour cells or micrometastatic nodal disease, and only 5% of patients had three or more positive sentinel nodes.

Another study not often as cited is the OTOASOR (The Optimal Treatment Of the Axilla—Surgery Or Radiotherapy) trial (2002–2009) after a positive sentinel lymph node biopsy in early stage breast cancer [52]. This was a randomized, single centre based out of the National Institute of Oncology Budapest, phase III, non-inferiority trial that published 8-year follow-up results, showing that axillary recurrence was 2.0% for cALND vs. 1.7% in their 50 Gy RNI arm (*p* = 1.00). OS at 8 years was 77.9% vs. 84.8% (*p* = 0.060), and DFS was 72.1% in cALND arm and 77.4% after RNI (*p* = 0.51). These results also show that RNI is statistically not inferior to the cALND treatment. Similar, OTOASOR allowed for mastectomy accounting for 16% of patients. Interestingly, 25% of patients in the cALND arm had pN1mi disease, but 22% had pN2a/3a disease.

In terms of toxicity, the risk of lymphedema as measured in arm circumference at one year in the Z0011 was 11% vs. 6%, *p* = 0.78 [53], while in the AMAROS it was 8% vs. 6%, *p* = 0.33 [51]. This is interesting given that the Z0011 had a much less extensive lymph node irradiation. The OTOASOR rates of (any clinical sign of) lymphedema, paraesthesia, swelling, arm pain, and shoulder mobility and were higher in the cALND group (15.3%) than in the RNI group at 1 year (4.7%). Of note, combining cALND and radiotherapy in the axillary treatment in the cALND arm further increased morbidity in those 18/57 patients (31.5%).

These trials have shown that patients with clinically negative axilla and positive sentinel lymph nodes have a risk of 27–33% of further involvement with ALND, which is comparable to the risk reported in the NSABP B-32 (26%) [8]. However, no axillary treatment or axillary RT was not associated with an excessively high nodal recurrence risk. Several questions were raised following these trials about the optimal RT fields and the role of tumour biology in LRR. Currently, we are awaiting the results of the BIG 2.04 MRC/EORTC SUPREMO trial (NCT00966888), which is testing the optimal radiation fields in the intermediate-risk group (chest wall only vs. no RT).

The Intergroup-Sentinel-Mamma (INSEMA) GBG75, ABCSG43 trial (NCT02466737), is a prospective, randomized trial comparing SLNB versus no axillary surgery in patients with early stage breast cancer (clinically/imaging T2N0) who are BCS candidates including WBI. The second phase, including patients with involved sentinel lymph nodes (1–3 macrometastases) randomized to either SLNB alone or completion ALND (protocol design 2011–2014 and patient enrolment from September 2015 to April 2019) [54], attempted to answer some of the questions, such as whether SLNBx can be avoided in patients having a mastectomy.

POSNOC (NCT02401685) is a randomized multicentre non-inferiority trial evaluating the role of axillary treatment (axillary node clearance or radiotherapy) in women with intermediate-risk breast cancer with one or two nodal macrometastases. All women will receive adjuvant systemic therapy (chemotherapy and/or endocrine therapy) and may receive breast or chest wall radiotherapy. A total of 1900 patients will be followed for 5 years to determine axillary recurrence. The study started in August 2014 and completion is expected in December 2026.

SLNB after neoadjuvant chemotherapy in patients with biopsy-proven positive axilla was attempted in several trials. A meta-analysis (2016) showed that the SLNB identification rate was 90% (95% CI: 87–93%), and the false-negative rate was 14% (95%CI: 11–17%), which was influenced by the number of lymph nodes retrieved. The false-negative rate is lower at 4% (0–9%) when ≥3 lymph nodes are retrieved, and when using the dual tracer (11%) vs. single tracer (19%) technique [55].

### 3.4. Radiotherapy

The question of whether regional nodal irradiation (RNI) is needed in intermediate-risk patients treated with ALND was addressed in two trials. The EORTC 22922/10925 (1996–2004) trial compared patients with low-intermediate risk disease to RNI of the supraclavicular fossa and the first three levels of the internal mammary chain (IMC) vs. no RNI. A small proportion of the population (8%) received radiation to the axilla. The 10-year LRR was 8.3% vs. 9.5%, respectively; RR was 2.7% vs. 4.2%, respectively; and DFS was significant in favour of RNI (72% vs. 69%; *p* = 0.04), yet OS was not significantly different (82.3% vs. 80.7%; *p* = 0.06) [56]. The National Cancer Institute of Canada (NCIC)- MA.20 trial (2000–2007) also attempted to address this question and included patients treated with BCS exclusively who had T2–T3 breast cancer with high-risk features, such as GIII, lymphovascular invasion, ER-negative tumours, or pathological N1. The randomization compared WBRT + RNI (supraclavicular fossa and IMC) to WBRT only. A total of 85% of patients had N1 disease. Full axilla was irradiated whenever ≤10 nodes retrieved in dissection or >3 nodes were positive. The rate of isolated 10-year regional recurrence was 0.7% vs. 2.7%, respectively. DFS was significantly different at 82% vs. 77%, *p* = 0.01, in favour of WBRT + RNI. However, OS was not different 82.8% vs. 81.8%, *p* = 0.38, respectively [57]. The key trials in intermediate-risk breast cancer can be seen in Table 2.

In terms of toxicity, the rate of lymphedema was higher for RNI in the EORTC 22,922 (12% vs. 10.5%) and MA.20 (4.5% vs. 8.5%). Pulmonary fibrosis was also higher for RNI in EORTC 22,922 (4.3% vs. 1.3%; *p* < 0.001), whereas the acute pneumonitis rate in the MA.20 was 1.2% vs. 0.2%, *p* = 0.01.

Advances in genomic expression profiling have allowed for more tailored treatment decision making. For example, the RxPONDER trial (2011–2017) showed no benefit of adjuvant chemotherapy among postmenopausal women with 1–3 positive lymph nodes who had an Oncotype DX RS ≤ 25 [58]. Further, a subset analysis of on-trial looking into Oncotype Dx (Genomic Health, Redwood, CA, USA) scores and LRR has shown that patients with N1 disease and a low Oncotype Dx score had a lower 10-year LRR (1.5% vs. 11%, *p* = 0.051) [59]. Currently, the TAILOR RT trial (NCT03488693) is ongoing with the goal to use molecular biomarkers to identify a subgroup of 1–3 axillary node positive breast cancer patients with ER+ biomarker low risk breast cancer (Oncotype DX RS ≤ 25) who could be spared RNI.

## 4. Locally Advanced, High Risk (T3N+, T4, N2–3)

### 4.1. Mastectomy

Historically, patients with locally advanced breast cancer were treated with mastectomy and ALND [60,61,62]. The EBCTCG meta-analysis (2014) showed that the 5-year LRR risk for T3–T4 disease was 36% [43]. The 10-year LRR without radiotherapy of disease with ≥4 positive nodes involved was 32.1% and BCSM was 67.3%. The benefit of mastectomy in locally advanced diseases extends up to patients 80 years or older receiving a modern-day treatment with a reduction in BCSM and improvement of OS [63].

### 4.2. Breast-Conserving Surgery

BCS may not be an option for some of the high-risk patient population because removing tumours to clear margins with an acceptable cosmetic outcome may not be feasible. However, BCS can be feasible after neoadjuvant chemotherapy. Approximately 1 in 4 women requiring mastectomy in the NSABP B-18 trial (1988–1993) became eligible for BCS after four cycles of Anthracycline-based chemotherapy [64]. Randomized trials including patients with Human Epidermal Growth Factor 2 (HER2)-positive or triple-negative disease have a higher conversion rate to BCS of approximately 4 in 10 [65,66]. The BCS conversion rates did not change with the addition of Taxanes to Anthracycline compared to anthracyclines alone in the NSABP-B27 trial (1995–2000) [31]. One reason for this might be that many patients accrued were eligible for BCS upfront. Further, the presence of suspicious calcification that rarely responds to chemotherapy and inability to assess tumour response in such scenarios might preclude BCS [67].

### 4.3. Axillary Surgery

Neoadjuvant chemotherapy (NAC) allows a less aggressive dissection with the potential of less morbidity in patients with clinically matted axillary lymph nodes. The NSABP B-18 and B-27 have mandated that patients have an axillary dissection after neoadjuvant chemotherapy. Those without complete response in lymph nodes had the highest locoregional recurrence risk at 10 years (17–22%) [68].

SLNB after neoadjuvant chemotherapy in patients with biopsy-proven positive axilla was attempted in several trials. The first was ACOSOG Z1071 (2009–2011), a phase 2 clinical trial in which 756 women with T0–4, N1–2, M0 breast cancer who received neoadjuvant therapy underwent SLNB followed by ALND [69]. Of these, 79% had mapping with the combination of radioactive colloid and blue dye. At least one SLN was identified in 93% of patients, 41% had a complete pathologic response, and in 21% of patients, the only node with disease was the SLN. The reported FNR was 12.6%, but it decreased to 10.8% when two tracers were used and 9.1% when more than two SLNs were obtained. When the clipped node was resected, the FNR fell to 6.8% [70].

The SENTINA study (2009–2012) enrolled 1737 women randomized to four arms. Arm A included cN0 patients who underwent SLNB prior to neoadjuvant chemotherapy. In arm B, in the SLN was positive (pN1), and a second SLNB was performed after chemotherapy. Women with cN+ lymph nodes underwent SLNB + ALND if they were rendered node-negative (arm C) after chemotherapy, or upfront ALND (arm D) if they were not. A total of 1022 women underwent SLNB and the detection rate was 99.1%. In arm B, the detection rate was 60.8% and the FNR 51.6%. In arm C, the detection rate was 80.1% and FNR was 14.2%. Mirroring ACOSOG Z1071, FNR rates decreased with number of nodes removed (7.3% with 3 SLN in arm C) and dual-tracer technique (8.6% in arm C) [71].

The Canadian SN FNAC trial (2009–2012) enrolled 153 patients with breast cancer (T0–3, N1–2). They underwent both SLNB and ALND after neoadjuvant chemotherapy. The axillary pathologic complete response rate was 34.5%. The identification rate was 87.6% and FNR was 8.4%. Single-agent mapping was associated with an FNR of 16.0% vs. 5.2% for dual-agent mapping. FNR also decreased with number of SLNs removed, at 4.9% for two or more SLNs [72].

Lastly, Targeted Axillary Dissection (TAD) (2011–2015) was described by Caudle et al. in 2016 [73]. It involves the removal of the SLN and the clipped node after neoadjuvant chemotherapy. A total of 208 patients with biopsy-proven nodal metastases prior to chemotherapy were enrolled and 191 underwent ALND. In patients who had SLNB with ALND, the FNR was 10.1%. When the clipped node was analysed, the FNR fell to 1.4%. Of note, the clipped node was not retrieved in 23%. TAD followed by ALND yielded an FNR of 2.0%.

We await the results of the MAC19 study (ALLIANCE A11202) (NCT01901094), which was a randomized Phase III trial evaluating the role of ALND in breast cancer patients (cT1-3N1) who still have positive sentinel lymph nodes after NAC. The trial began in February 2014 and has since completed accrual.

Based on the aforementioned data, CCO and ASCO guidelines recommend SLNB using the dual-tracer technique and retrieving the clipped node, after neoadjuvant chemotherapy [74].

### 4.4. Radiotherapy

Post-mastectomy radiotherapy (PMRT) trials [60,61,62] that ran in the 1980s, which included patients with high-risk disease showed that PMRT reduced LRR by 27%, improved DFS by 18% and prolonged OS by 9%. However, those trials were criticized for the low numbers of retrieved axillary lymph nodes on axillary surgery with a subsequent unusually high axillary recurrences, a potential understaging of patients with N2 as an N1 disease, and the use of less effective systemic options (tamoxifen for a year or CMF chemotherapy) [75]. The EBCTGC meta-analysis (2014) showed that PMRT reduced 10-year LRR (32.1% vs. 13%; *p* ≤ 0.00001) and reduced BCSM (67.3% vs. 61.1%; *p* = 0.04) for disease with ≥4 positive nodes [43].

In the neoadjuvant setting, the NSABP-B18 and B27 did not include patients with skin or chest wall involvement (T4) or fixed or matted lymph nodes (N2). However, T4 and N2 diseases were allowed in the ACOSOG Z1071 trial. The benefit of radiotherapy was evaluated and after a median follow-up of 5.9 years, and the LRR for the entire population in that trial that also included patients with intermediate risk was 6.1%. Receiving RT was associated with a significantly lower LRR on multivariable analysis [76].

Parallel to the MAC19 trial, we also await the results of the NSABP B-51/RTOG 1304 (NCT01872975) randomized Phase 3 clinical trial that evaluated the role of regional nodal irradiation in patients with biopsy-proven axillary nodes prior to neoadjuvant chemotherapy who subsequently converted to ypN0. It was initially slow to accrue following activation in July 2013, but has since also completed accrual.

## 5. Metastatic Disease

Four RCTs looked into whether locoregional treatment (LRT) would improve outcomes in patients with de novo metastatic disease. The Tata Memorial trial (2005–2013) compared LRT vs. no LRT. Chemotherapy was assigned and started prior to randomization for patients with unresectable primary tumours. Patients with a single metastatic site amenable to curative therapy were excluded. Only 25% of the population had metastasis to ≤3 sites. LRT improved locoregional progression-free survival (LRPFS), but was associated with a detrimental effect on distant progression-free survival (DPFS). The 2-year OS was 41.9% vs. 43%, respectively [77].

The MF07-01 trial (2007–2012) made a comparison with upfront LRT with subsequent systemic treatment (ST) vs. primary ST. Almost half (47%) of the population had a single metastatic site. The 5-year OS was 41.6% vs. 24.4%, *p* = 0.005, in favour of the LRT arm. Patients with hormone receptor-positive (*p* = 0.01), HER2-negative (*p* = 0.01), patients younger than 55 years (*p* = 0.007), and solitary bone metastasis (*p* = 0.04) had a significant benefit from upfront LRT [78].

The Eastern Cooperative Oncology Group (ECOG)–The American College of Radiology Imaging Network (ACRIN) E2108 trial (2011–2015) randomized women who had de novo metastatic disease with no progression after 4–8 months of systemic treatment to LRT vs. ST. LRT reduced 3-year locoregional progression (16.3% vs. 39.8%; *p* ≤ 0.001). Free margin after surgery was associated with a significantly lower hazard of locoregional progression, while RT was not. The 3-year OS was not different (68.4% vs. 67.9%; *p* = 0.57) [79]. Unlike the MF07-01 trial, OS was not different in patients with any receptor status or in patients with oligometastatic disease.

The Austrian Breast and Colorectal Cancer Study Group (ABCSG)-28 POSYTIVE trial (2011–2015) made a similar comparison to the MF07-01 and allowed LRT in the ST arm on progression. The number of metastatic sites was not reported. After a median follow-up of 3.1 years, LRT did not impact LRPPS (*p* = 0.88) with no subset of patients found to have a significant benefit from LRT. The median survival was 2.9 years in the LRT arm vs. 4.6 years in the ST arm. Interestingly, this trial allowed RT to metastatic sites [80].

In contrast to locoregional management in the metastatic setting, a landmark trial shifting the paradigm and focus to treating oligometastatic sites in patients with controlled primary tumour (defined as at least 3 months after primary tumour treated definitively with no progression) has recently shown OS benefit. The SABR-COMET trial (2012–2016) compared stereotactic body radiotherapy (SBRT) to oligometastatic sites vs. standard of care. Approximately 25% of included patients in the trial had breast cancer in origin. The 5-year OS was significantly different in the SBRT arm compared to the standard of care arm (42.3% vs. 17.7%; *p* = 0.006.) [81].

Currently, the NRG-BR002 study (NCT02364557) is focused exclusively on newly oligometastatic breast cancer and testing whether treating metastases with surgery or high-dose radiation improves survival. This Phase IIR/III trial of standard of care therapy with or without SBRT and/or surgical ablation activated in December 2014 and temporarily stopped in 2019 while awaiting signal to convert to the phase III component. Future research in this realm provides an exciting opportunity to explore advanced technology to work synergistically with systemic treatment.

## 6. Conclusions

An overview of the evidence to support the role of surgery and radiotherapy was presented for all stages of breast cancer. Understanding the timeline of evidence might assist in appreciating the current context leading to modern day treatment recommendations. Oncologists are strongly encouraged to accrue patients in ongoing trials to evolve our understanding of breast cancer and optimize the therapeutic index of locoregional treatment.

## Figures and Tables

**Table 1:** Trials for low-risk breast cancer.

Research Question	Trial	Accrual Period	Control Arm	Comparison Arm	Age	Tumours	Tumour Size	Receptor Status	Median FU	LRR	OS
Omission of WBRT in BCS	CALGB 9343	1994–1999	WBRT	No WBRT	≥70	Invasive	≤2 cm	ER+ (100%)	12.6 Years	2% vs. 10% *	67% vs. 66%
	PRIME II	2003–2009	WBRT	No WBRT	≥65	Invasive	≤3 cm	ER+ (100%)	7.3 Years	1.4% vs. 13.1% *	81% vs. 80.4%
APBI in BCS	NSABP B39-RTOG 0413	2005–2013	WBRT	APBI (3DCRT/Brachytherapy)	≥18	Invasive + DCIS	≤3 cm	ER+ (80%)	10.2 Years	3.9% vs. 4.6%	91.3% vs. 90.6%
	Ontario RAPID	2006–2011	WBRT	APBI (3DCRT)	≥40	Invasive + DCIS	≤3 cm	ER+ (90%)	8.6 Years	2.8% vs. 3.9%	94.3% vs. 93.6%
RT dose for WBRT	Ontario	1993–1996	50 Gy	42.56 Gy	≥18	Invasive	≤5 cm	ER+ (73.9%)	10 Years	6.7% vs. 6.2%	84.4% vs. 84.6%
	UK START_B	1999–2002	50 Gy	40 Gy	≥18	Invasive	≤3 cm	NR	9.9 Years	5.5% vs. 4.3%	80.8% vs. 84.1% *^,^**
	FAST_Forward	2011–2014	40 Gy	26 Gy, 27 Gys	≥40	Invasive	≤5 cm (98%)	ER+ (70%)	6 Years	2.3% vs. 1.5% ***	94.6% vs. 94.4%

Abbreviations: WBRT, Whole Breast Radiotherapy; BCS, Breast Conserving Surgery; APBI, Partial Breast Irradiation; RT, Radiotherapy; Gy, Gray; 3DCRT, 3D Conformal Radiotherapy; DCIS, Ductal Carcinoma in Situ; ER, Estrogen Receptor; NR, Not Reported; FU, Follow-up; LRR, Locoregional Recurrence; OS, Overall Survival. * Values in bold represent a significant difference; ** Converted from All-Cause Mortality; *** Result for 40 Gy vs. 26 Gy arm only.

**Table 2:** Key trials in the intermediate-risk breast cancer group.

Research Question	Trial	Trial Accrual	Control Arm	Comparison Arm	Tumour Size	Nodal Status	Receptor Status	Systemic Treatment	Median FU	LRR	RR	DFS	OS
ALND vs. no ALND	Z0011	1999–2004	ALND	No axillary treatment	≤5 cm	Macro: 62–55%	ER+ (83%)	Overall: 7% CTX: 58%, ET: 67%	9.25 Years	6.2% vs. 5.3%	0.5% vs. 1.5%	NR	NR
	AMAROS	2001–2010	ALND	Axillary RT	≤5 cm	Macro 59–62%	NR	Overall: 90% CTX: 61% ET: 79%	6.1 Years	NR	0.4% vs. 1.19%	86.9% vs. 82.7%	93.3% vs. 92.5%
Regional RT vs. no regional RT	EORTC 22,922	1996–2004	Local	Local + RNI	Any (96.5% ≤5 cm)	N1: 43% N2–3: 13%	ER+ (73.5%)	Overall: 85% CTX: 55% ET: 60%	10.9 Years	9.5% vs. 8.3%	4.2% vs. 2.7%	**69%** **vs.** 72% *	80.7% vs. 82.3%
	MA.20	2000–2007	WBRT	WBRT + RNI	Any (99% ≤5 cm)	N1: 85% N2: 5%	ER+ (75%)	Overall: 91% CTX: 86% ET: 76%	9.5 Years	6.8% vs. 4.3%	2.5% vs. 0.5%	**77%** **vs.** 82% *	81.8% vs. 82.8%

Abbreviations: ALND, Axillary Lymph Node Dissection; RT, Radiotherapy; WBRT, Whole Breast Radiotherapy; RNI, Regional Nodal Irradiation; Macro, Macrometastasis; N, Nodal; ER, Estrogen Receptor; CTX, Chemotherapy; ET, Endocrine Treatment; NR, Not Reported; LRR, Locoregional Recurrence; RR, Regional Recurrence; DFS, Disease-Free Survival; OS, Overall Survival. * Values in bold represent a significant difference.
